# Discrete time information diffusion in online social networks: micro and macro perspectives

**DOI:** 10.1038/s41598-018-29733-8

**Published:** 2018-08-08

**Authors:** Jinshan Qi, Xun Liang, Yi Wang, Hengchao Cheng

**Affiliations:** 1 School of Information, Renmin University of China, Beijing, 100872 China; 2 School of Computer Science and Technology, Huaiyin Normal University, Huai’an Jiangsu, 223300 China

## Abstract

Opinions shared publicly in online social networks spread broadly and at an extremely high speed. However, modelling information diffusion in online social networks is still a challenge that is intriguing to many researchers. To monitor public opinions online, it is necessary to model the process of information dissemination. In this paper, we first study information diffusion based on the network structure and time occupation. By taking into consideration the availability of a user, e.g., his online or offline state, we present the discrete-time bi-probability independent cascade model. We next analyse the information diffusion from a macro perspective. A diffusion model is established by merging the interferences from other events and the cumulative effect that occurs over time. Finally, we observe the factors in online social networks that impact a message’s diffusion from a micro perspective and discuss more complex user behaviour and various types of interferences with their effects from a macro perspective. Experiments are conducted with real world data, and the experimental results justify our models.

## Introduction

In the age of we-media, the popularity of the Internet and the emergence of instant communication platforms have changed the mode and fashion of information diffusion, leading participants to be more private, pervasive and autonomous than ever before. In platforms such as Twitter, Facebook, We-chat and forums, users who contribute the main stream of information diffusion not only passively receive information but also actively create and disseminate messages, greatly enlarging the breadth, depth speed of news propagation.

Events occurring in the real world usually motivate related messages that spread on the Internet, and sometimes messages even disseminate with explosive speed. A large number of messages cause shocks to the public. These shocks sometimes exist for a short time, while others may persist for a long time because of a variety of factors. Among these shocks, some spread quickly and widely and have big influences. To avoid public panic, the diffusion of certain information with extreme ideas should be monitored and controlled. Therefore, the problem of online information diffusion has gained considerable attention in the field of online social networks.

The independent cascade model (IC) and Linear Threshold model (LT)^1^ are the two classic methods for depicting the diffusion of influence in online social networks, which explain the diffusion from the perspectives of probability and threshold, respectively. Both methods have been widely cited and developed since their proposal. Sauti^2^ proposed the asynchronous independent cascade model (AsIC), where time was emphasized during the process of diffusion. Wan^3^ used the method of maximum entropy to define a probability involved in the model and then analysed the probability. Chen^4^ developed a weighted cascade model where the infective probability of the successor node is the reciprocal of its in-degree. A new diffusion model combining user de-emphasis, garbage user filtering and reading probability was recently proposed in^5^. Pathak^6^ presented a generalized LT (GLT) based on LT and simulated a multi-level cascade diffusion process. He^7^ designed a competitive LT (CLT) and used it to find the most influential groups. More LTs were developed later, and many of them focused mainly on optimizing the algorithm by maximizing the impact. To control the spread of false information in time, Iouliana^8^ established a dynamic LT (DLT) and extracted an optimization problem to recognize the set of users who are most likely to transfer the information. Taxidou^9^ proposed a model for information provenance and diffusion, and thus improved from the W3C PROV Data Model. The structural, sociological and temporal aspects were taken into consideration in^10^, and real-time analysis methods on social media regarding information diffusion were investigated simultaneously. Pfitzner^11^ proved that betweenness preference is present in empirical temporal network data and it influences the length of the shortest time-respecting paths. Cheng^12^ found that temporal and structural features are key predictors of cascade size involving breath and initially. By observing independent cascades of the same content, the largest cascade can be predicted even though the cascades differ greatly in size. Halberstam^13^ found that information reaches like-minded users more quickly and users are disproportionately exposed to like-minded information by date from Twitter. By using a technique which combines automatic topic extraction and sentiment analysis^14^, addressed news diffusion regarding Brexit in the UK on Facebook, and performed an analysis on massive users interacting with Brexit^15^ used data on frequencies of bi-directional posts to describe the dynamic relationships in Facebook datasets, and investigated the internal structure of the networks. Other scholars used machine learning techniques to find the properties of the diffusion process. For instance, Lahiri^16^ found that the genetic algorithms were able to improve the diffusion model based on simulations of a large and rich class of diffusion models for online social networks.

When faced with specific application problems, information content, network structure, and time occupation may play important roles. For example, the key users can be divided into structure-dominant users, content-dominant users, and time-dominant users. The content-dominant users are influential because they publish authoritative content on online social networks. For a structure-dominant user, the key user may be determined by its own characteristics as follows: its strengths associated with other nodes, its location in the path of information diffusion, or the location in a community that it belongs to. A typical time-dominant user is the one with an online social network addiction. Clearly, a user’s online state determines whether he has access to the information. However, few studies have considered this prerequisite. Additionally, an accurate evaluation of the influence among users is the basis for the subsequent diffusion process, but the existing literature always assumed the probability as known when conducting experiments involving their proposed models. The current information diffusion models focus on the cascade and analyse the results solely from the view of structure instead of content and time. The content and the source of the messages also have a big impact on the diffusion, yet models seldom take control of these variables.

To overcome these shortcomings, the nodes’ states of staying online and time occupation are studied by extending the IC model into a discrete-time bi-probability independent cascade model. To determine the influence from users, the expectation maximum (EM) method is employed in our paper. The Monte Carlo method was used to simulate the diffusion process, and we extract several important factors and build a macroscopic diffusion equation based on the results of the simulation. Finally, we perform a numerical simulation considering factors such as the effects of herding, time accumulation and interferences from other events.

## Empirical Results

Here we give the results of the information diffusion of SINA microblogging according to our models proposed. The details of the data and models are presented in the section Materials and Methods.

### Experiments on the diffusion of a single message

#### Diffusion probability between users

We calculate the probabilities of infectivity between users *p*_*v*,*w*_ using the proposed EM method. Since *P* is set to be a constant to simplify the problem, we first compare the differences of {*p*_*v*,*w*_} in response to the variation of its values.

**Figure 1:**
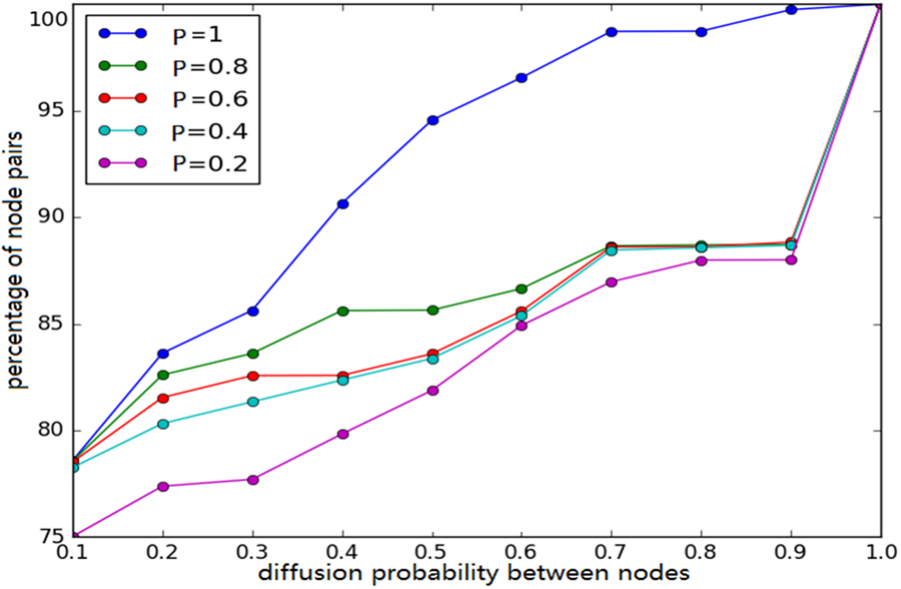
The distribution of weights between users with different *P*. The *x*-axis denotes the diffusion probability between nodes and the *y*-axis indicates the percentage of node pairs.

Figure 1 shows the difference of probability distribution, where the *x*-axis denotes the probability of activation between users and the *y*-axis indicates the percentage of pairs. A higher *P* value indicates that users are more likely to remain on the microblogging platform and spend more time on it. As seen from Fig. 1, the probability distribution appears to be relatively distinct only when *P* is at the extremes (minimum value 0.2 and maximum value 1). When the value of *P* is intermediate, the difference is very small and almost negligible. Therefore, we set the P value as 0.5 in subsequent simulations.

In the extreme cases, we found that approximately 80% of the node pairs have an infecting probability less than 0.1, while nearly 10% of the node pairs have an infecting probability more than 0.9, or are almost completely affected. Probabilities with *P* equalling 1 are significantly higher than other conditions in our experiments, and the main difference lies in the value between 0.4 and 0.9. Obviously, when *P* equals 1, it is consistent with the traditional models without the validity probability. When the *P* value is less than 1, the polarization trending is more serious. In other words, most users have a small probability of spreading information, and some users may spread information from some others all the time, with a small part of node pairs spreading information occasionally. We believe that the probability distribution is consistent to the structure of the user network. Generally, the density and relationships of users in microblogging platforms are not high, which means that most of the network is sparse. Therefore, most node pairs have very low influences, with few communications. However, some small communities still exist where users have close relationship with each other, thus the infiltration of information is very deep. The influence among them is nearly 100%, which means a user would propagate almost every message that his parent node releases.

#### Results of the diffusion

**Figure 2:**
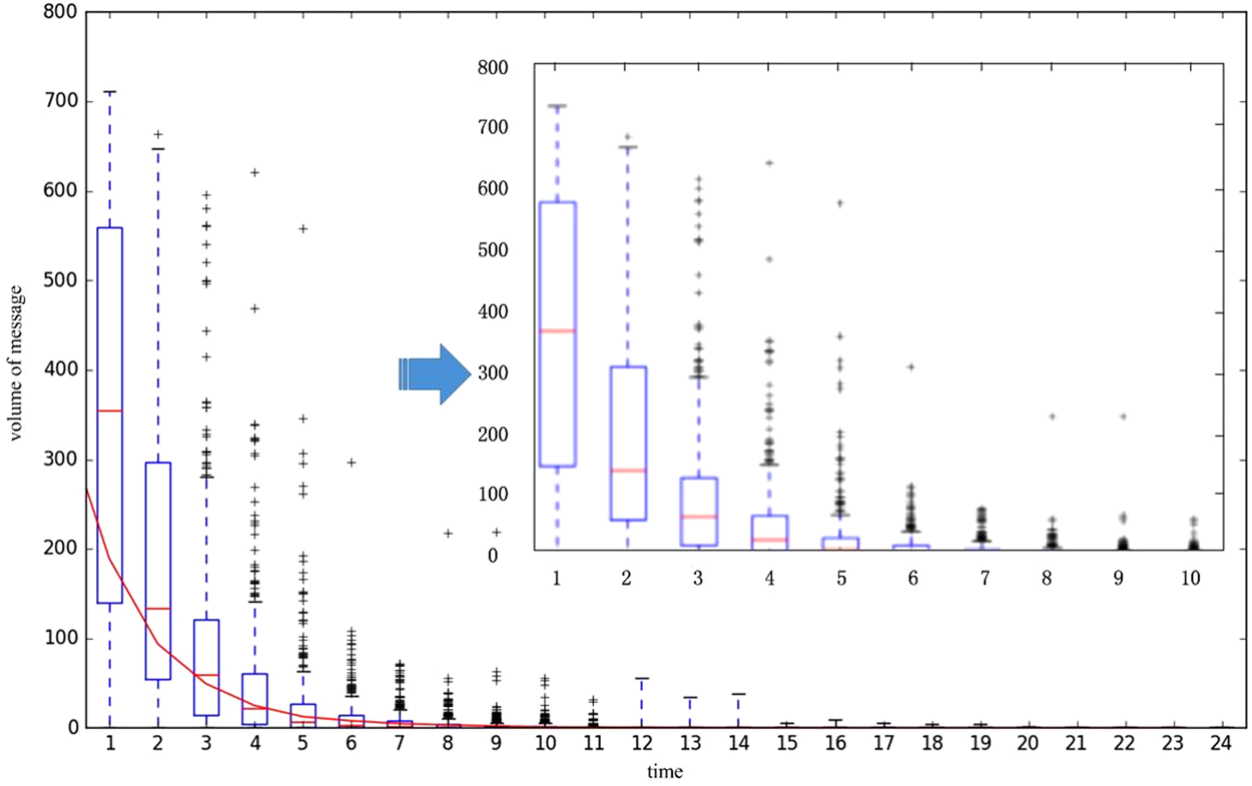
Diffusion speed of 5000 runs by the Monte Carlo method. The *x*-axis denotes the time, and the *y*-axis denotes the number of messages generated.

Based on the diffusion probabilities above, we conducted our simulation of Model I 5000 times. The results of our simulations are shown in Fig. 2, where the *x*-axis represents the timespan since the information appeared and the *y*-axis indicates the number of messages generated. We use box-plot to describe the distribution of propagation in each period. The top small dash is the top edge of the simulation results, representing the maximum amount of diffusion in the experiment; the upper edge of the box represents the upper quartile of the propagation in the simulation, and the lower edge represents the lower quartile. While the middle horizontal line represents the median of the propagation.

From Fig. 2 we see that large amounts of dissemination occur in the first 10 timespans, while the speed rapidly declined afterwards. Moreover, the red small dashes represent the median value of speed, which tends to fall quickly, as do the quartile values. Additionally, we tried to fit the curve of median speed over time, and the fitting function is *y* = 448.61e^−0.54*x*^ with *R*^2^ = 0.986, demonstrating the trend of exponential decay. This result highlights the importance of time as the spreading process ends in only several timespans, which is helpful to us for governing public opinions online. If one wants to control public opinion, actions should be taken in the effective period because the proliferation may finish in no more than 10 hours. This conclusion is one of the values of Model I.

Yang^17^ developed a K-Spectral Centroid (K-SC) clustering algorithm, and found six typical patterns of information diffusion. These patterns differ in the number of peaks, the sharpness of speed descending, the time when a peak appears, and the peak values. We reduced the factors to the peak numbers and the time of the first peak to simplify the classification. Based on the different morphological characteristics, the diffusion patterns were divided into four types, i.e., delay and multi-peaks, delay and single-peak, instant and single peak, and instant and multi-peaks.

**Figure 3:**
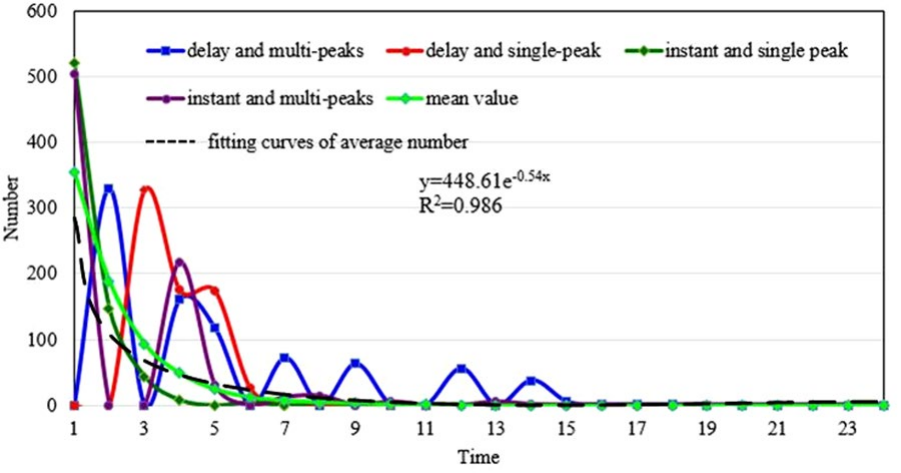
Different types of information dissemination over time where the *x*-axis represents time and the *y*-axis represents the amount of diffusion.

Figure 3 shows the four typical diffusion patterns and the average speed over time, where the *x*-axis represents time and the *y*-axis represents the amount of diffusion. We found that the pattern of the instant and single-peak is most similar to the global average diffusion pattern. Both peaks had a large amount of diffusion at the beginning, but the speed decayed rapidly with time. Moreover, although the probability of multi-peaks is relatively high, the subsequent peaks are small fluctuations. This also reflects the importance of controlling the first outbreak. Their characteristics and amount are shown in Table 1.

**Table 1:** More detailed descriptions of the four patterns of information diffusion.

Type	Characteristics		Percentage
	Burst time	Number of peaks	
1	instant	1	33%
2	Delay	1	6%
3	instant	>1	46%
4	Delay	>1	15%

We see that over 80% of the information disseminates soon after the information appeared, which indicates the importance of the initial shock. In addition, the number of single-peak patterns is slightly less than multi-peaks patterns, indicating that the information is prone to spring back throughout the diffusion. From the perspective of the number of peaks, the single-peak pattern is weaker than the multi-peaks pattern, showing that shocks tend to have bounds during its lifetime, though the gap is not very large.

Next, we set up three comparative experiments to test the impact of the initial node on information diffusion. To some degree, the amount of a user’s fans is the symbol of its strength. Some studies have proved that the ingress of nodes in the social network follow the power-law distribution. In our discussion, we rank the number of fans, and regard those whose fans more than 90% as the strong node, less than 10% as weak node and the middle is as a medium node. We use the number of affected users, the life cycle, and the coverage to evaluate the spread of information. In detail, the number of affected users refers to the number of users who propagated the message in the experiment, the life cycle refers to the duration from when the message is released to the end of the message, and the coverage is defined as the number of affected users divided by number of fans. It can be seen from Table 2 that the initial node with a higher in-degree can generate more spreading. When the initial node has few in-links, it is less likely to spread information widely. In another word, users preferred to retweeting information about events from celebrities or groups. Therefore, the influence of ordinary users is small.

**Table 2:** The results of diffusion from different types of nodes.

		Strong nodes	Medium nodes	Weak nodes
Followers	Total number	34922	281	30
Number of affected users	Average number	742.489	97.867	0
	Std. error	67.761	0.148	0
Lifecycle	Average number	10.288	16.521	0
	Std. error	3.354	3.950	0
Coverage rate	Average number	0.021	0.148	0
	Std. error	0.002	0.176	0

It is interesting to find that both strong and weak nodes have shorter life cycles than the intermediate ones when spreading information. The reasons may be as follows. First, strong nodes are more likely to track an event and update messages with a higher frequency when faced with emergencies, so the attraction of a single message will quickly decay. Second, strong nodes of the event can also be divided into two categories: one is with strong correlations and the other is in weak relation. For a node in the first category, its neighbours would respond to the messages in a short time, whether forwarding or commenting. In the case of a node with weak correlations in the second category, there is only a very small probability for its neighbours to obtain and forward the information published by it, which, consequently, shortens the lifetime.

Intermediate nodes have the largest coverage rate, while weak nodes have the smallest coverage. As analysed before, strong nodes only have some links with large weight, thus the probability of an effective impact is very small. With a large base, the overall coverage is reduced. For intermediate nodes, it is more likely to have a strong cluster where information propagates from one to another more efficiently, leading to high coverage. For the weak nodes with low activity, there is a smaller audience size and a weaker influence, so it is more difficult to disseminate the information.

Another point is that the standard deviations of these indicators are small, which means that information released by the same user with the same quality can spread out to a similar scale. Although the different diffusion processes differ from each other, each one of them shows some inherent trends in the lifecycle, speed and coverage rate. This also provides a basis for the supervision of public opinion.

Moreover, we also conducted experiments on variation of quality of information for a specific topic. When the information itself has a high quality, which means that messages are more attractive and important, information is more likely to be spread. In this paper, we take strong nodes as an example to show the results of information with different qualities in Table 3.

**Table 3:** Diffusion of strong nodes with different information.

Quality	Average time	Average number of diffusion	Average rate of coverage
0.9	23.671	34929.735	0.836
0.7	23.405	2425.935	0.058
0.5	23.045	1415.535	0.034
0.3	22.705	1085.340	0.026
0.1	22.430	1078.930	0.026

Table 3 shows that only when the quality of information is as high as 0.9 can the amount of information diffusion be extremely large. Otherwise, even if the quality is relatively high, the coverage is still below 5%; and when the information is not attractive enough, the coverage will be very low.

From the above simulation results, we have obtained the following conclusions. First, our method for solving the probability with the EM algorithm is more reasonable than using content similarity between the node pairs in online social networks. Second, discrete time bi-probability IC matches well with the diffusion process of information in the real world. Third, actions should be taken in the first 10 hours to control the spread of information. Otherwise, the delay is too late for the government to gain control. Fourth, in the process of monitoring public opinions, we should focus on two kinds of groups: one is the strong nodes with strong links, and the other is the small-scale networks with strong connections. Fifth, when the source of diffusion is constant, the overall effect of the diffusion will have some regularity. For example, the entire information distribution is consistent with the exponential distribution, and the overall impact scale is almost the same. Sixth, for a small number of messages with high information quality, they need to be controlled quickly from their source.

### Experiments on the diffusion of messages from a macro perspective

#### Experiments of events with different beginning times

Assuming that only a single event occurs in the user network. The event signal can be represented as *f*(*τ*) = *βτ*^−1.5^. We first study the diffusion of information under this ideal situation, where information of the event will not be covered by other events in a very short time and can be fully spread.

**Figure 4:**
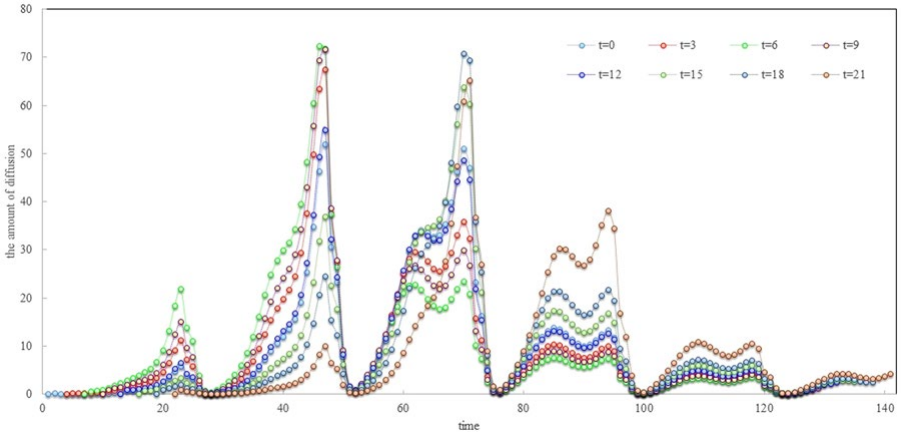
Spreading of events occurred at different times with number of 2000 and no interferences where *x*-axis represents time and *y*-axis represents the amount of diffusion.

Since the time when an event occurs varies widely, we make a quantitative analysis of the effect caused by the initial time of an event. In the section of Material and Methods, we have analysed the actual activity of users in the network. We find that the user’s active cycle is approximate 3 hours, and the user’s activity has a great influence on the diffusion of information. Therefore, 3 hours can also basically distinguish the difference in the spread of public information at the initial stage. In the experiment, we took three hours as the time unit to perform the analysis so that important times with a large number of active users are covered. Eight groups of contrasting experiments were performed and their results are demonstrated in Fig. 4.

In the graph, the *x*-axis represents the time and the *y*-axis represents the amount of diffusion. It can be seen that the event occurs around *t* = 6 and will produce the maximum amount of diffusion in the first day of diffusion. This is because that the user becomes active from this moment, and messages can be forwarded as soon as the event occurs. At the same time, there still exists some time before the moment when users are mostly active. Therefore, when the best breaking times occur, it already has a certain accumulation of communication users, which laid the foundation for the rapid dissemination of the message. For events with an earlier outset, the quality of information quickly decays because it cannot spread effectively in the early stages. That is, why the effect of information diffusion will be worse. For events with a later outset, though it will spread rapidly in the network at the beginning, the impact weakens because of the lack of a sufficient user basis. Additionally, though events occurring at different times have different speeds, their diffusion patterns are generally consistent and are in accordance with the users’ behaviour patterns.

Ideally, the time that an event occurs affects the maximum speed of information diffusion, and events that occur at approximately 6:00 and 18:00 have a greater speed of diffusion as well as the speed of volatility. While for an event that occurred at approximately 0:00, the speed of diffusion is relatively stable at a low level. From the average speed events, events that occur after 15:00 are more likely to have higher speed.

It is worth noting that in the real world, information cannot achieve such a complete diffusion to all the users. Generally, the life cycle of information diffusion related to an event is very short, and the number of affected users relates closely to the quality of information and other factors.

#### Experiments of events with different volumes of information

**Figure 5:**
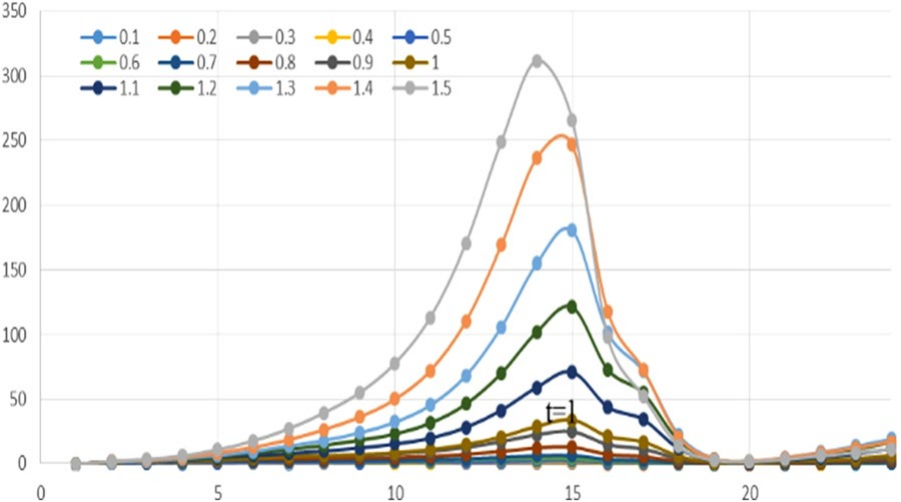
The first stage of the event’s spreading with different information. The number of nodes is 2000 in the experiment, and there is no interference. Here, the *x*-axis represents time and the *y*-axis represents the amount of diffusion.

As the quality of information with different events differs greatly from each other, the diffusion effect will also be greatly affected. Although all event signals are treated as one unit at the very beginning, they would be scaled by the volume of information *β* as *f*(*t*)*β*. We analyse the effects of *β* on the information diffusion process. Since different stages have similar diffusion patterns and the first is the most typical one, we show the first stage of experimental results in Fig. 5.

In Fig. 5, the *x*-axis indicates the time and the *y*-axis indicates the number of users that have received the information. It is shown that when *β* ≤ 1, the amount of information diffusion in a short time is very small. That is, for a general event, the amount of diffusion in the most effective time is very limited. When *β* > 1, the diffusion of information will be significantly improved, and the time it takes to reach the maximum will occur relatively early. Considering the quality of information will also affect the life cycle of the information (the greater the quality of information has a longer life cycle). It is speculated that messages of events with a high volume of information will spread much faster and further than messages of ordinary events.

#### Experiments of events with different interferences

**Figure 6:**
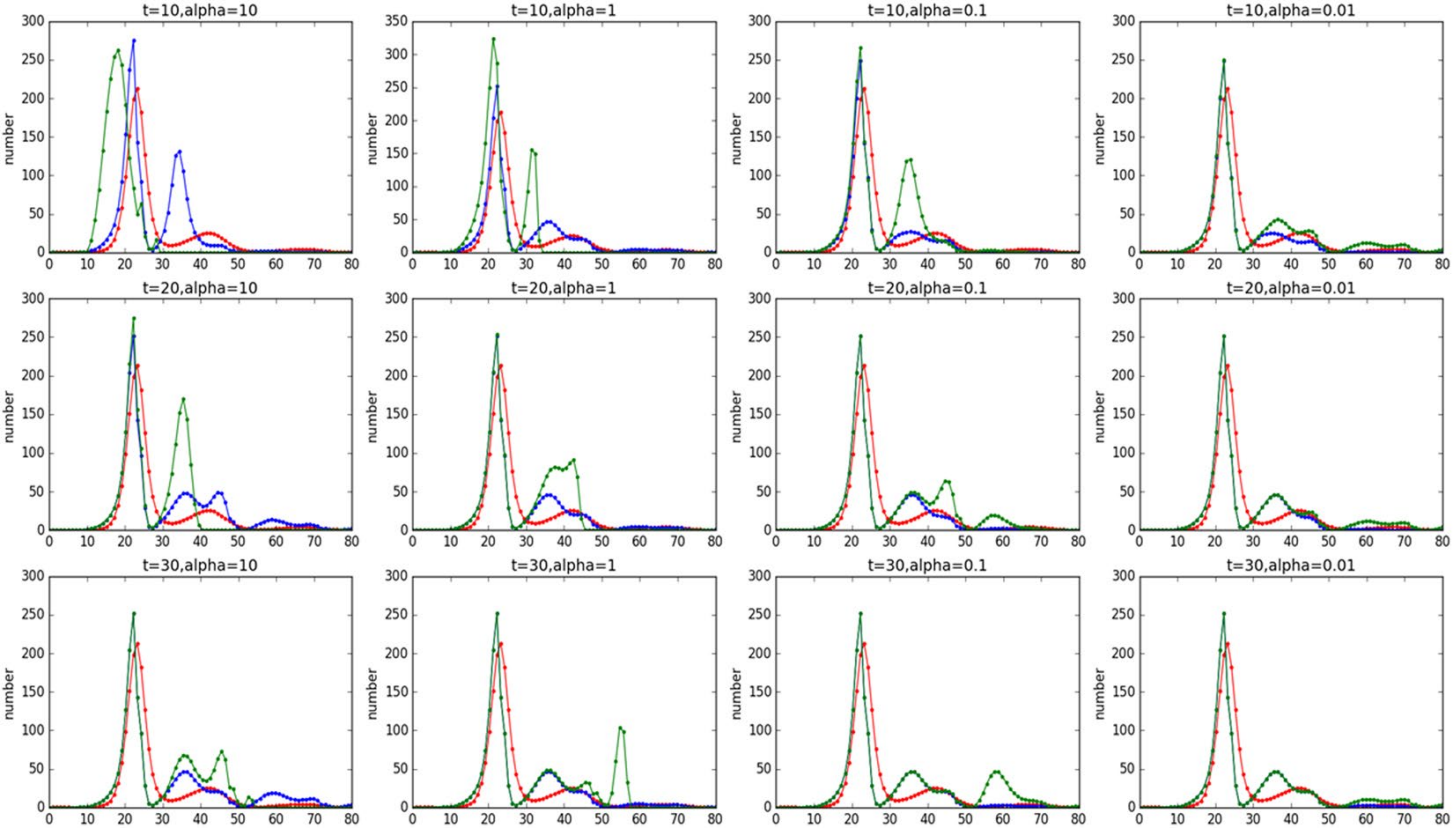
Spreading of messages associated with an event with regard to different times when interference occurs, different degrees of influence and different forms of interference. The *x*-axis represents time, and the *y*-axis represents the number of users who have forwarded the message.

Suppose only two events exist that have influence on each other in the system, with event signals A and B representing them separately. Here, we only analyse the diffusion of the first event. Three groups of experiments were carried out with different disturbing times. Within each group, the association strength according to Definition 5 was considered. Interferences of these two signals occur in two ways: time dependent and time independent ones, corresponding to Definitions 7 and 10. According to Definition 7, the signal depends only on the influence of the current time, or the signal is time independent. While Definition 10 means that the previous influence still have an impact on the signal, or the signal is time dependent. The results of the experiments are shown in Fig. 6.

In Fig. 6, the *x*-axis represents time, and the *y*-axis represents the number of users who have forwarded the message. It is assumed that the *α*, defined in Definition 5, of these two events remains unchanged. Additionally, the red line represents the original diffusion of the event in the absence of interference; the green line represents the diffusion of the time-independent interference occurring at time *t*; and the blue line represents the diffusion of the time-dependent interference occurring at time *t*. Each row represents a set of experiments with consistent time of disturbances, and each column represents a set of experiments with a consistent degree of impact.

First, from the degree of overlap of the graphics, we found that differences in the first group of four experiments are larger than the other two groups. In others word, the closer the two events occur, the greater the impact. When *α* > 1, the mutual promotion between the events helps them have greater diffusion in a shorter period of time. When *α* < 1, the smaller alpha indicates more serious competition. Additionally, the weak side will be significantly lower than the normal level of diffusion. Second, the influences in diffusions are divided into time-dependent and time-independent types. The effect of the time-dependent type is more obvious than that of time-independent type, and the influence is also related to the intensity of time and influence. Third, there is no doubt that the more that *α* deviates from 1, the greater the effect it causes.

#### Experiments with real world data

**Figure 7:**
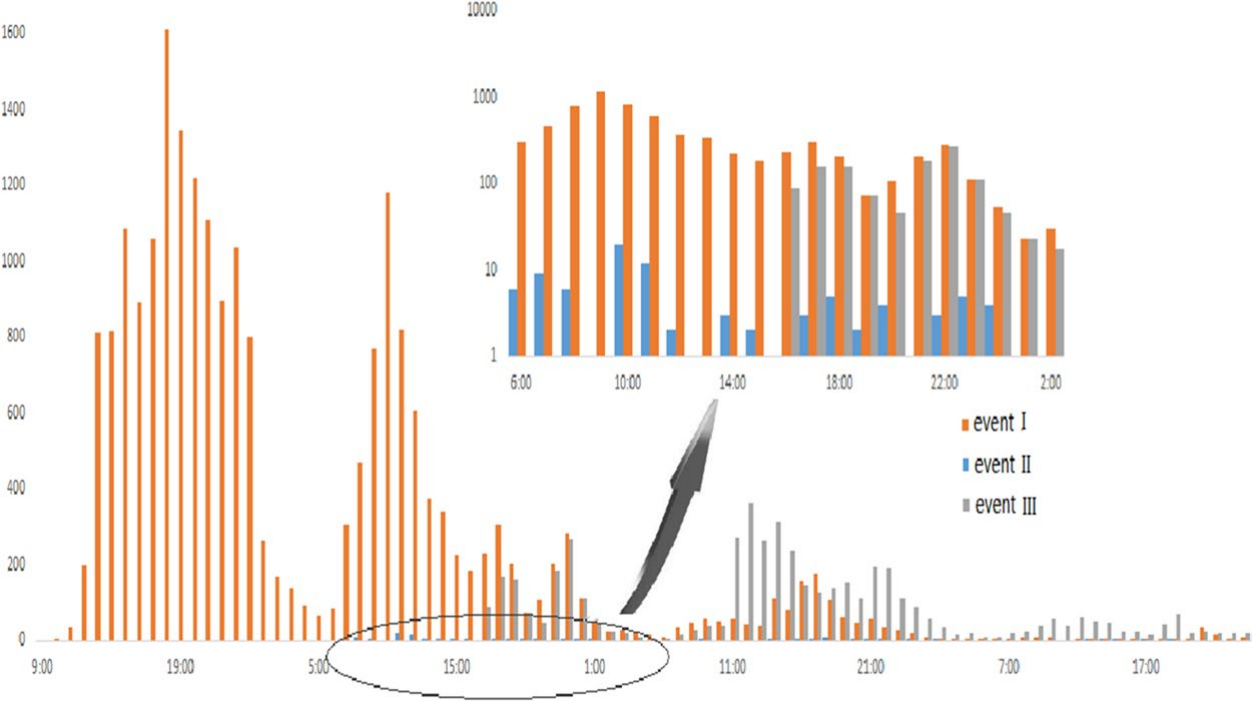
Spreading of three events in SINA microblogging from 2016-12-24 to 2016-12-29. The *x*-axis denotes time and *y*-time denotes the number of diffusions.

Next, we collected data from the real world to analyze the information diffusion. “High rebate in the drug field,” “High consumption of officers” and “Anti-vice in Beijing” are all about social events with higher attention, and can be considered to contain the same amount of information. To simplify the narration, we call them event I, event II and event III, respectively. Their corresponding signals are denoted as signal A, signal B and signal C. We collected actual data of these three events from the SINA microblogging platform and the results obtained are shown in Fig. 7.

It can be seen from Fig. 7 that event I received much attention beginning when it occurred, especially on the first day. Thus *f*(*t*) of signal A is strong at the beginning and the backward difference ∇*f*(*t*) is small. On the next day, it followed the dissemination trend of the first day, but in the afternoon, it encountered the impact of signal B. However, because signal B itself is not strong, its impact on signal A is small and the value ∇*f*(*t*) is still small, which means that the influence caused by *r*_B,A_(*t*) is not large enough. Then, the third day, the spread of event A decayed rapidly, which causes ∇*f*(*t*) to increase correspondingly. Both the decay of signal A and the interference from signal C led to the decrease. Apparently, event C affected the diffusion of event A more than event B, indicating that *r*_C,A_(*t*) > *r*_B,A_(*t*). Then, we analyse the diffusion from the perspective of signal B. It should have had a greater diffusion because of the rich information. However, due to the strong interferences from signals A and C, the diffusion is almost negligible. This is also consistent with the result in the simulation experiments: The closer two events occur, the greater the amount of interference they would have on each other. The experimental results reveal the importance of correlated functions among signals and time of interferences simultaneously.

## Discussion

To understand information diffusion in online social networks in the age of we-media, in this paper, we depicted the process and characteristics of information diffusion from both the micro and macro perspectives.

The microcosmic part studies a specific user and the influence among users. The Monte Carlo method was employed to simulate the diffusion process. We found in the experiments that most of the dissemination finished within the first 10 hours, with a trend of exponential decay. A large variety of different morphological characteristics, such as multi-peaks, single-peak, mild and fierce, appeared during the experimental processes of diffusion. Additionally, when the structure of the network is deterministic, we can analyse the characteristics of the network, focusing more on the strong nodes and their close relationships with each other. The information contributed by a small group of users with close relationships also needs to be monitored so that the diffusion of information can be more effectively controlled.

We then analysed the information diffusion from a macroscopic perspective, trying to explore its diffusion pattern over time. A numerical function was additionally established with the factors of information volume, peak time of an event, user activity, probability of forwarding, interferences from outer platforms and other events as well as noise to depict the changes of speed over time. In the process of modelling, herd behaviour and interferences from other platforms as well as from other events were discussed. The analysis of data collected from SINA microblogging verified the validity of our simulation results. The findings help us to investigate the key factors in information diffusion, as well as their effects in the process.

Our research is consistent and connects the difference of the diffusion with a single message and the whole event, supporting the monitoring of diffusion from both the microscopic and macroscopic perspectives. In future work, we will focus on the quality of information with different types of events and develop predictive methods for early warning and control based on the quantitative analysis.

## Material and Methods

### Data

To reduce the impact of some of the main factors, such as topic and resource, we collected data about information spreading about the *2015 Tianjin Explosions* 1^1^from SINA microblogging for 10 days (originally delivered by TOP NEWS). This dataset consists of 41783 users and the routes of information dissemination, among which we analyse the influence relations between users.

### Information diffusion models in online social networks

In the following models are built to describe the mechanism of information diffusion over time in online social networks, from which we extract some significant factors. A numerical model is also established in this section to perform quantitative analysis as well.

### Diffusion model from a micro perspective

The discrete-time bi-probability independent cascade model is proposed to depict the diffusion process of information over time in online social networks.

An online social network is represented by a directed graph *G* = (*V*, *E*), where nodes in *V* stand for users in the network and links in *E* represent the relationship between users. For ∀*v*, *w* ∈ *V*, *v* ≠ *w*, *e* = (*v*, *w*) denotes the link from *v* to *w*, which means that information can be transmitted from *v* to *w*. Node *v* in *G* is denoted by *F*(*v*), the set of nodes with an incoming link to *v*, which can be understood as a set of child nodes of *v* as follows: *F*(*v*) = {*w*: (*v, w*) ∈ *E*}. Similarly, we define *B*(*v*) as a set of parent nodes of *v* as follows: *B*(*v*) = {*u*: (*u*, *v*) ∈ *E*}.

We call nodes active if they have been infected, and we also assume that nodes can only switch from inactive to active. The basic elements in our model are similar to the classical IC. However, in our model, the state of each node involves two different aspects: behaviour and availability. On one hand, nodes can be divided into active and inactive states, depending on whether they have an operation (e.g., comment, reply or forward) on a specific message. When a user forwards the message, then the state of the user changes into the active state. However, if the user does not forward the message, then it stays inactive. On the other hand, a user’s state of availability can also be divided into online and offline, which determines this access to the information.

In the real world, a user gets online to gain information during timespan [*t*_*i*−1_, *t*_*i*_) (*i* = 1, 2, 3, …) with a certain probability, called available probability *k*_*u*_ ∈ ***R*** for node *u*. When a user is offline, the corresponding node is unavailable; and when a user is online, the corresponding node is available. Only when a user is online can he receive the information. In other words, the availability of a node is the basis of achieving and forwarding information. It is worth noting that there is no need to consider the online state of a user if he has been infected, indicating that the node’s state of behaviour plays a more important role than availability. For each directed link (*u*, *v*), we specify a real value *k*_*u*,*v*_ (0 < *k*_*u*,*v*_ < 1) as the diffusion probability.

**Figure 8:**
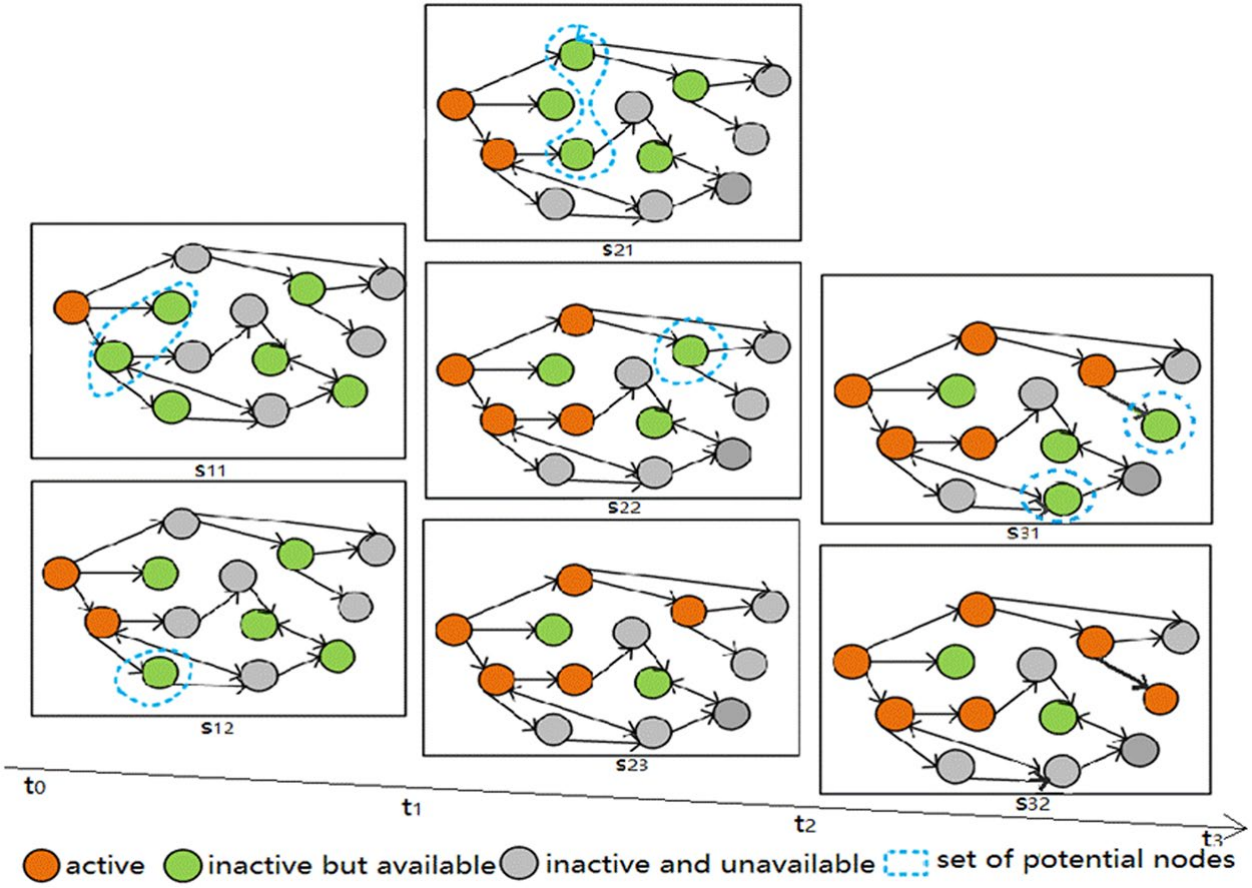
An example of information diffusion with 13 nodes in a network and the changing state of nodes over time. The whole process has three timespans, within which several cascades occur. The first and third timespans have two cascades, while the second has three cascades. Within every cascade, the information spreads for two steps: child nodes that are available at this time form the potential node set; and then the active node attempts to infect the potential nodes.

Figure 8 shows the diffusion of information in a given network, where orange stands for active nodes, green represents online yet inactive nodes, and grey indicates that the user is offline and inactive. The given network has 13 nodes with three different states, i.e., active, inactive but available, inactive and unavailable. For the simple network in Fig. 8, the information spreads in three timespans. Both timespans [*t*_0_, *t*_1_) and [*t*_2_, *t*_3_) have two cascades, while [*t*_1_, *t*_2_) has three cascades. Within every cascade, the information spreads in two steps: the child nodes that are available at this time form the potential node set, where the activation process will occur in the next step; then the active node attempts to infect the potential nodes. At *s*_11_ during [*t*_0_, *t*_1_), there is just one active node. The others are available nodes whose child nodes form a potential node set. The active node attempts to infect its child nodes in the set at *s*_12_. As a result, one node is infected successfully and becomes active, while the other one fails and remains inactive. This is the final state of time *t*_1_ and then the message reaches [*t*_1_, *t*_2_), with three cascades. For each cascade in this timespan, the active nodes repeat the two steps as in the former cascade. Similarly, the third timespan [*t*_2_, *t*_3_) has two cascades where nodes’ states change accordingly. Finally, six nodes become active and the process is complete.

**Figure 9:**
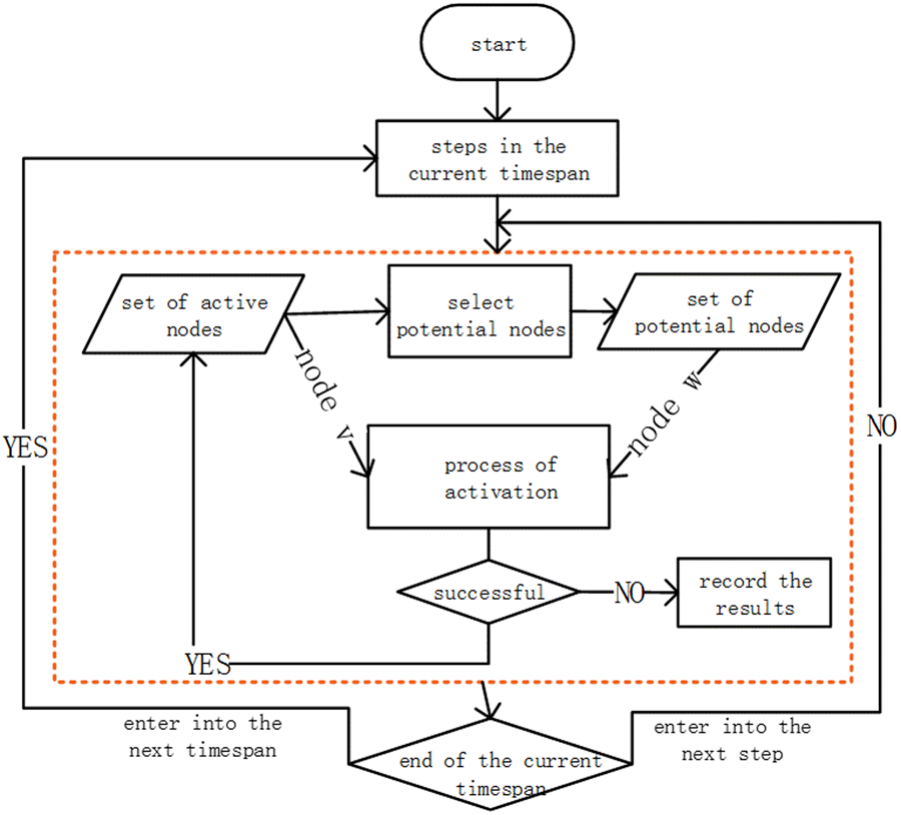
The information diffusion process in a timespan.

Figure 9 reveals the process of information diffusion in a given timespan [*t*_*i*−1_, *t*_*i*_], *i* = 1, 2, 3, … Because the time is discrete and cascades can occur more than once in one timespan, we denote the possible cascades within [*t*_*i*−1_, *t*_*i*_] as step(*i*), *i* = 0, 1, 2, 3, … The set of active nodes in the *j*th step in [*t*_*i*−1_, *t*_*i*_) is denoted as *D*(*t*_*i*_, *s*_*i*,*j*_). The diffusion of information proceeds from an initial set of active nodes, denoted by *D*(*t*_1_, *s*_11_). In the *j*th step in timespan [*t*_*i*−1_, *t*_*i*_), we select active nodes that have active child nodes and group them into a potential set. Then, node *v* from the active set has a single chance to infect his child node *w* in the potential set with probability *p*_*v*,*w*_. If *v* succeeds, *w* will become active in the next step, shown as follows:

1 $$w\in \{\begin{array}{c}D({t}_{i},{s}_{i,j+1}),{s}_{i,j} < {\rm{step}}(i)\\ D({t}_{i+1},{s}_{i+1,1}),{s}_{i,j}={\rm{step}}(i)\end{array}$$

In the case where multiple parent nodes of *w* become active, their activation attempts are sequenced in an arbitrary order in the same step of a timespan. Once *v* has tried to infect *w*, it would not be able to infect it in the subsequent steps. The process terminates when no possible activation exists, or the observation time runs out.

The process described above can be summarized in Model I.

#### Model I

- Given an original set of active nodes *D*(*t*_1_, *s*_11_) and let *D*(*t*_*i*_, *s*_*i*,*j*_) denote the set of active nodes of the *j*th cascade in timespan [*t*_*i*−1_, *t*_*i*_), *i* = 1, 2, 3… Every node stays online at a certain probability, and each pair has its own infection probability.
- Information spreads up to step(*i*) cascades in timespan [*t*_*i*−1_, *t*_*i*_), *i* = 1, 2, 3…
- For each level of dissemination, once a node *v* is active, all its adjacent nodes that are inactive and online are likely to become active. If a potential active node *w* has more than one active neighbour, they would attempt to infect *w* arbitrarily. After *w* has been infected, it joins the active node set as in formula (3).
- Once *v* has tried to infect *w*, it has no chance to try again.
- The whole process ends when it runs out of the valid time or no potential nodes exist.

Model I depicts the diffusion process over time in online social networks by incorporating the state of a user as online or offline, the probability of infection among nodes, and the relations of time occupations in the cascades. The Monte Carlo method can be used to depict the influence of probability.

As mentioned before, the diffusion probabilities between nodes are fundamental in our experiments. In this part, the probability is first deduced according to the diffusion mechanism described in Model I. Then, we develop a method of calculating *p*_*v*,*w*_. To simplify the process of analysis, we consider timespans rather than cascades within each timespan.

Table 4 shows the main symbols in the analysis of diffusion probabilities below. Using *r*_*w*_(*t*) to denote the probability that node *w* is infected at time *t*. Subsequently, *r*_*w*_(*t* + 1) is expressed as follows:

2 $$\begin{array}{rcl}{r}_{w}(t+1) & = & 1-\prod _{v\in B(w)\cap {A}_{w}(t)}({k}_{w}(1-{p}_{v,w})+(1-{k}_{w}))\\  & = & 1-\prod _{v\in B(w)\cap {A}_{w}(t)}(1-{k}_{w}{p}_{v,w})\end{array}$$

Here, the condition that node *w* is not infected by *v* at time *t* consists of two scenarios: node *w* is inactive with probability 1 − *k*_*w*_, and node *v* fails to infect node *w* although *w* is active with probability *k*_*w*_(1 − *p*_*v*,*w*_).

**Table 4:** Main symbols and definitions in analysing probabilities.

Symbol	Definition
*p*_*v*, *w*_	probability that node *w* is infected by *v*
*k*_*w*_	probability that node *w* gets online
*r*_*w*_(*t*)	probability that node *w* is infected at time *t*.
*C*(*t*)	set of all the active nodes by time *t*
*D*(*t*)	set of nodes newly becoming active at time *t*
*R*_*w*_(*t*)	set of nodes that have attempted to infect *w* by time *t*
*A*_*w*_(*t*)	set of nodes that are able to infect node *w* at time *t*
*D*_*s*_	an information diffusion episode *s*
*t* ^( *s*)^	time *t* in an episode *D*_*s*_
*r*_*w*_^( *s*)^	probability that node *w* becomes active in an episode *D*_*s*_
*S*_*v*, *w*_^+^	set of episodes that satisfies *v* ∈ *A*_*s*_(*t* + 1) and *w* ∈ *D*_*s*_(*t* + 1)
*S*_*v*, *w*_^−^	set of episodes that satisfies *v* ∈ *A*_*s*_(*t* + 1) and *w* ∉ *D*_*s*_(*t* + 1)
*N*	number of nodes (users) in the given network
*n*	time, *n* = 0, 1, 2, …
*U*(*n*)	number of un-informed users
*B*(*n*)	number of infected users
Δ*B*(*n*)	number of users getting infected at time *n*
*f*(*n*)	function of quality of information at time *n*
*q*_A,B_(*t*).	the influence one signal would have on the other at time *t*
α(*t*)	the influence of these two signals at time *t*
*β*	quality of information
*S*(*n*)	volume of external shocks at time *n*
*n*_*b*_	the time an event occurs
*ε*	noise in the process

Let *D*(*t*) be the set of nodes newly becoming active at time *t*. Then, the information diffusion episode is the union of ordered sets *D* = *D*(0) ∪ *D*(1) ∪ … ∪ *D*(*T*). Let *C*(*t*) denote a set of all the active nodes by time *t*, and *R*_*w*_(*t*) the set of nodes that have attempted to infect *w* by time *t*. Then, *C*(*t*)\*R*_*w*_(*t*) denotes the set of nodes that are able to infect node *w* at time *t*, recorded as *A*_*w*_(*t*). It is unable to determine when situations such as (a) *v* ∈ *A*_*w*_(*t*) and *w* ∈ *D*(*t* + 1), or (b) *v* ∉ *C*(*t*) occur because we cannot get valid information about the link (*v*, *w*). Therefore, the probability of a known diffusion result can be expressed as,

3 $$L(\theta ;D)=(\prod _{t=0}^{T-1}\prod _{w\in D(t+1)}{r}_{w}(t+1))(\prod _{t=0}^{T-1}\prod _{v\in D(t)}\prod _{w\in F(v)\backslash C(t+1)}(1-{k}_{w}{p}_{v,w}))$$

Let { *D*_*s*_: *s* = 1, 2, …, *S*} denote a set of *S* independent information diffusion episodes, and we can define the following objective function with respect to the diffusion probability set *θ* = { *p*_*v*,*w*_}.

4 $$L(\theta )=\sum _{s=1}^{S}\mathrm{log}\,L(\theta ;{D}_{s})=\sum _{s=1}^{S}\sum _{t=1}^{{T}_{s}-1}(\sum _{w\in {D}_{s}(t+1)}\mathrm{log}\,{r}_{w}^{(s)}+\sum _{v\in {D}_{s}(t)}\sum _{w\in F(v)\backslash {C}_{s}(t+1)}\mathrm{log}(1-{k}_{w}{p}_{v,w}))$$

5 $${r}_{w}^{(s)}=1-\prod _{v\in B(w)\cap {A}_{w}({t}^{(s)}-1)}(1-{k}_{w}{p}_{v,w})$$

Here, *r*_*w*_^(*s*)^ stands for the probability that node *w* becomes active in an episode *D*_*s*_, and it is defined in (5). The term *k*_*w*_ denotes the available probability in Model I.

Next, we need to find the most likely diffusion probability to maximize (4). Since it is difficult to get the maximum value by solving the partial derivative functions or adopting the gradient rising algorithm, we apply the EM algorithm to solve the problem for *θ*.

For node *v* ∈ *A*_*w*_(*t*), if *w* ∈ *D*_*s*_(*t* + 1), then the attempt though link (*v*, *w*) succeeds with probability ${\widehat{k}}_{v,w}/{{\widehat{r}}_{w}}^{(s)}$ and fails with probability $1-{\widehat{p}}_{v,w}/{{\widehat{r}}_{w}}^{(s)}$. Here, ${\widehat{p}}_{v,w}$ forms the current estimate set $\widehat{\theta }=\{{\widehat{p}}_{v,w}\}$. Considering all these cases, we define a *Q*-function for *S* episode in (8),

6 $$\begin{array}{c}Q(\theta |\widehat{\theta })=\sum _{s=1}^{S}\sum _{t=0}^{{T}_{s}-1}\sum _{v\in {A}_{s(t)}}(\sum _{w\in F(v)\cap {A}_{s}(t+1)}(\frac{{\widehat{k}}_{v,w}}{{\widehat{r}}_{w}^{(s)}}\,\mathrm{log}\,{k}_{w}{p}_{v,w}+(1-\frac{{\widehat{k}}_{v,w}}{{\widehat{r}}_{w}^{(s)}})\mathrm{log}(1-{k}_{w}{p}_{v,w}))\\ \,\,\,\,+\sum _{w\in F(v)\backslash {C}_{s}(t+1)}\mathrm{log}(1-{k}_{w}{p}_{v,w})\end{array}$$

It is worth noting that we set the value of *k*_*w*_ to a fixed constant *P* without considering the difference of nodes and time. Meanwhile, the influence of different values of *P* on the probability is analysed in the following experiments. To obtain the optimized result, we set the condition ∂*Q*/∂*p*_*v*,*w*_ = 0, then

7 $${p}_{v,w}=\frac{1}{{\rm{P}}(|{S}_{v,w}^{+}|+|{S}_{v,w}^{-}|)}\sum _{s\in {S}_{v,w}^{+}}\frac{{\widehat{p}}_{v,w}}{{\widehat{r}}_{w}^{(s)}}$$

Here, *S*_*v*,*w*_^+^ stands for a set of episodes that satisfies *v* ∈ *A*_*s*_(*t* + 1) and *w* ∈ *D*_*s*_(*t* + 1), and *S*_*v*,*w*_^−^ differs from *S*_*v*,*w*_^+^ in the condition that *w* ∉ *D*_*s*_(*t* + 1). |*S*| indicates the size of set *S*.

### Diffusion model from a macro perspective

In a macro perspective, we need to predict the range and impact of the dissemination after a shock occurs. For this purpose, we treat users as a group rather than focus on a specific user. Thus, we analyse the information diffusion process by modelling the main factors from a macro perspective.

#### Model II

1. A number of initial seed nodes appear with the advent of an event, which thereby affects a large number of inactive nodes in the network.
2. Within each timespan [*t*_*i*−1_, *t*_*i*_), *i* = 1, 2, 3…, active users affect a portion of the inactive users with a certain probability. This probability is associated with the quality of information as well as users’ activity.
3. Other platforms may produce external stimuli to the diffusion of information, positively or negatively correlated with the amount of information diffusion in the previous timespan.
4. Uncontrollable factors exist during the diffusion process causing noise.
5. The whole process ends when the valid time runs out.

In Model II, more factors are extracted from the macro perspective to model the number of users who spread relative information. Next, we explain the factors in detail, as well as their influence during the process.

The quality of information is used to depict the strength of motivating users to forward the information, which is valued subjectively by the source, content and form of expression. Additionally, we introduce the event signal to describe the quality of information over time.

#### Definition 1 (Event signal)

An event signal is the strength of quality of information that changes over time and can be mathematically represented as a function of time.

Every event signal is treated as one unit at the very beginning. The intensity of a signal at time *t* is denoted by *f*(*t*). Through the definition of an event signal, we are able to have the specific expression or specific value over time, which is beneficial to studying the impact of other factors on the spread of public opinion.

#### Property 1 (Randomness of event signal)

The randomness of an event signal means that the strengths of signals are unpredictable, but follow a power-law falling trend.

Random signals are defined as the strengths of an infinite indexed collection of random variables {*X*(*t*): *t* ∈ *T*}. The strength of a relevant public opinion signal is uncertain, but from the empirical experiments, its time series shows a downward trend following the power law, and the current strength affects the strength in the future.

#### Property 2 (Discrete of event signal)

The relevant event signals are defined only in discrete times and have little practical meaning at other times.

Since the model of information diffusion in this paper is deduced in discrete time, we assume that the relevant public opinion signals also occur in discrete time. In the meantime, the interference among relevant events is very complex, making it difficult to use a unified function to express their mutual influences. As a result, event signals are random.

#### Definition 2 (Unit step event signal)

The unit step event signal is denoted by *ε*(*t*) = 1, if *t* > 0, and *ε*(*t*) = 0, otherwise.

If the unit step event signal jumps at *t* = *t*_0_, it can be expressed by *ε*(*t* − *t*_0_). It demonstrates the influence caused by the event at time *t*_0_, and the value remains constant. In fact, the impact of the event varies randomly over time, and thus, the signal can be expressed as *f*(*t*)*ε*(*t*) where *f*(*t*) indicates the influence of event.

#### Definition 3 (Unit impulse event signal)

The unit impulse signal is denoted by $$\delta (t)=\{\begin{array}{cc}0 & t\ne 0\\ \infty  & t=0\end{array}$$ with ${\int }_{0}^{\infty }\delta (t)dt=1$. Similarly, if the impulse occurs at time *t*_0_, it is denoted by *δ*(*t* − *t*_0_).

The reason for using an impulse function to represent the changes of a signal is that an event occurs suddenly with a shock to the public. Although its instantaneous influence is very large, the total energy of the whole life cycle can be regarded as one unit with an integration.

The relationship between the unit step event signal and the unit impulse event signal can be derived from the definition as follows: $\delta (t)=\frac{d\varepsilon (t)}{dt}$ and $\varepsilon (t)={\int }_{-\infty }^{t}\delta (\tau )d\tau$.

#### Definition 4 (Superposition of event signal)

The superposition of an event signal is the sum of two signals at time *t*. It can reflect the total intensity of the two signals.

#### Definition 5 (Associated strength of two event signals)

Associated strength of two event signals which is the influence one signal would have on the other at time *t*, denoted as *q*_A,B_(*t*).

We can define it as *q*_A,B_(*t*) = α(*t*)*f*_2_(*t*), where *f*_2_(*t*) denotes signal *B* and α(*t*) represents the relationship of these two signals. When α(*t*) > 1, then the influence at time *t* is further expanded. Conversely, when α(*t*) < 1, the influence at time *t* is reduced. Otherwise, when α(*t*) = 1, the influence at time *t* is the same as signal B. Obviously, *q*_A,B_(*t*) ≠ *q*_B,A_(*t*).

#### Definition 6 (Correlation function of event signals)

The correlation function *r*_A,B_(*t*) is the set of associated strength with *k* timespans at time *t*, which can show the influence of signals B to A in the whole process.

At time *t*, the correlation function is represented as *r*_A,B_(*t*) = {*q*_A,B_(1), *q*_A,B_(2), …, *q*_A,B_(*k*)}.

#### Definition 7 (Scaling of event signal)

The strength of event signal A is affected by another related signal B, and the strength at time *t* is denoted by *f*(*t*)*q*_A,B_(*t*).

#### Definition 8 (Difference of event signal)

The difference of the event signal represents the variation of the signal strength per unit time at time *t*, and can also be regarded as the rate of change of the event signal strength of the discrete signal at time *t*. The first-order forward difference of the event signal at *t* is defined as Δ*f*(*t*) = *f*(*t* + 1) − *f*(*t*) and ∇*f*(*t*) = *f*(*t*) − *f*(*t* − 1) is the first-order backward difference of *f* (*t*) at time *t*.

#### Definition 9 (Accumulation of event signal)

The event- signal accumulation operation is defined as $y(k)=\sum _{n=0}^{k}\,f(n)$, which describes the event signal over the entire effective time range.

#### Definition 10 (Convolution sum of two event signals)

There are two event signals with mutual influences. Signal A is expressed as *f*_1_(*t*), and signal B as *f*_2_(*t*). The correlation functions between them are expressed as *r*_A,B_(*t*) and *r*_B,A_(*t*) respectively, then the function of signal A is expressed as:

8 $${f}_{1}(t)\ast {r}_{A,B}(t)=\sum _{n=0}^{\infty }{f}_{1}(n){r}_{A,B}(t-n)$$

and signal B comes out as

9 $${f}_{{\rm{2}}}(t)\ast {r}_{B,A}(t)=\sum _{n=0}^{\infty }{f}_{2}(n){r}_{B,A}(t-n)$$

where * represents the convolution operation. It is worth noting that *f*_1_(*t*) * *r*_A,B_(*t*) ≠ *f*_2_(*t*) * *r*_B,A_(*t*).

The correlation function of signals B and A can be decomposed into a series of impulse functions, each of which has an influence on signal A. Since the influence has a time delay, the whole impact can be shown as the sum of all the impulses. This process helps the signal to change more smoothly and therefore can be denoted as convolution sum.

Definition 11 (Convolution sum of multiple event signals): When a number of event signals that have mutual influences on each other appear at the same time, their effects on signal A should be amassed to represent the total influence. Suppose that *N* signals whose impacts on signal A are *r*_1_, *r*_2_, …, *r*_*N*_. Then, the value of signal A would be affected by them as *f*(*t*)*r_1_(*t*)*…* r_*N*_(*t*).

It reveals that more than one event signals have isolated effects on a specific event at the same time.

#### Property 3 (Distribution law of convolution sum)

The property can be represented as *f*(*t*) * [*r*_1_(*t*) + *r*_2_(*t*)] = *f*(*t*) * *r*_1_(*t*) + *f*(*k*) * *r*_2_(*k*), where *f*(*k*) is the original signal, *r*_1_(*t*) and *r*_2_(*t*) represent different event signals which have an effect on the original signal.

When event signals affect the original one consistently, the associated signals can be summed in advance, and then convolved with the original event signal. The final influence can also be seen as a sum of the two signals that are superimposed on each other.

#### Property 4 (Combination law of convolution sum)

The property can be represented as [*f*(*t*) * *r*_1_(*t*)] * *r*_2_(*t*) = *f*(*t*) * [*r*_1_(*t*) * *r*_2_(*t*)], where *f*(*t*) is the original signal, and *r*_1_(*t*) and *r*_2_(*t*) represent different signals that have effects on the original one independently.

When two event signals A and B affect the original one independently, it can be regarded as being affected by signal A first, followed by signal B. The total influence can also be seen as the convolution of the original signal and a synthetic signal, where the synthetic signal is calculated as the convolution of signals A and B. This law reflects the property of disorder of the influence of independent opinion signals.

#### Property 5 (Time shifting of convolution sum)

If *y*(*t*) = *f*(*t*) * *r*(*t*), then *f*( − *m*_1_) * *r*(*t* − *m*_2_) = *y*(*t* − *m*_1_ − *m*_2_).

This property reflects the influence of the convolution sum when the time of the event signals changes.

With the definitions above, we have depicted the quality of information and interferences from other related events. Given a network of *N* nodes, an event occurs at the birth-time *n*_*b*_ with *S*_*b*_ users who immediately forward the messages about the event. Other users can receive these messages through their connections and become a propagator with a certain probability. The changes of a user’s activity in a day are almost the same, which is in accordance with his behaviour in a day.

To simplify Model II, we make the following assumptions: (1) Every user can only participate once during the whole procedure. (2) There is only one source of the whole event, which means that nothing else can affect the diffusion.

This model attempts to establish a relation among the number of new propagators Δ*B*(*n*) and the parameters *n*, *N*, and *β*. Just as in Model I, a node has two states of *U* and *I*, where *U* and *I* represent the states of the uninformed and informed users, respectively. *U*(*n*) is the number of users who stay uninformed in time *n*. Consequently,

10 $${\rm{\Delta }}B(n+1)=U(n)\sum _{t={n}_{b}}^{n}({\rm{\Delta }}B(t)+S(t))f(n+1-t)+\varepsilon $$

11 $$U(n+1)=U(n)-{\rm{\Delta }}B(n+1)$$

where Δ*B*(0) = 0, *U*(0) = *N*. It is obvious that $B(n)=\sum _{t=0}^{n}{\rm{\Delta }}B(t)$ and *B*(*n*) + *U*(*n*) = *N*. Additionally, information from different platforms can influence each other through diversions and communication on the Internet, thus an external shock *S*(*n*) is added to denote the influence from an outer stimulation in time *n*. The number of *S*(*n*) can be defined as follows,

12 $$S(n)=\{\begin{array}{ll}{S}_{b}, & n={n}_{b}\\ a\,\mathrm{log}(1+{\rm{\Delta }}B(n-1)), & n\ne {n}_{b}\end{array}$$

Here, higher diffusion has a positive effect in the next time span, so a large number of users in the network are likely to spread the same information during the same time span, which forms herd behaviour.In the model above, some terms are explained here:

- Δ*B*(*n*) + *S*(*t*) stands for the summation of the newly informed users of the same platform and outer shocks.
- The function of outer shock *S* is self-adaptive. At the birth time *n*_*b*_, *S* is exactly the number of original infected users. We assume that *S* is related to the diffusion later with a delay. It is based on the assumption that a large amount of information spreading is associated with high quality, so other platforms are likely to have a larger impact because of the same reason. In contrast, the outer shock decreases with a smaller amount of diffusion. To avoid the tremendous difference in dissemination numbers, we take the logarithm of the original value and add a constant to adjust it. Further, Δ*B*(*t* − 1) + 1 is used to ensure that it works even if Δ*B*(*t* − 1) equals 0.
- All the active users are able to affect inactive users at time *n*. That is, why we add all the active users from different times and different platforms together.
- The number of newly informed users can be represented by the product of available targets *U*(*n*), infected users and the strength of the event signal *f*(*n* + 1 − *t*).
- A noise term *ε* of a very small value is introduced into our model to depict the unpredictability of the environment online.

Moreover, the pattern of information diffusion is related to the behaviour of users, and we take the periodicity of the users’ activity into consideration,

13 $${\rm{\Delta }}B(n+1)=A(n+1)(U(n)\sum _{t={n}_{b}}^{n}({\rm{\Delta }}B(t)+S(t))f(n+1-t)+\varepsilon )$$

**Figure 10:**
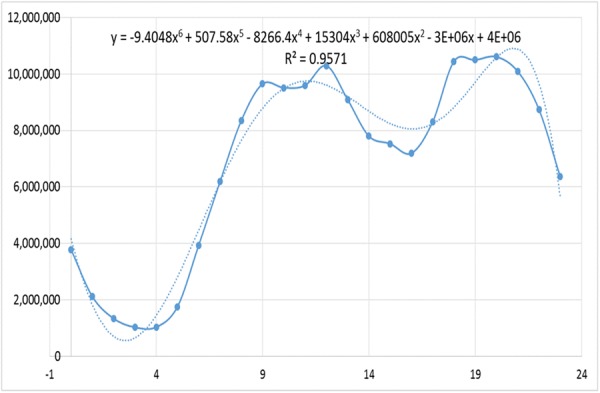
Number of active users in a day from *Tencent News*. The *x*-axis denotes the time in a day and the *y*-axis indicates the average number of active users.

Here, *A*(*n*) is a periodic operator representing the degree of user activity. We collect data on the user’s active daily hours from *Tencent News* 2^2^for a month, and the average daily number of active users is shown in Fig. 10.

It can be seen from Fig. 10 that the users begin to be active at 7:00 in the morning. The peak of activity occurs at 13:00 and 21:00, which is consistent with the daily behaviour of the users. Usually, people have lunch and take a break in the afternoon, while they begin to relax at 21:00 at the end of a day’s work before sleeping. By fitting the data, we obtain the function as *y* = −0.4048*x*^6^ + 501.6*x*^5^ + 15304*x*^4^ + 608005*x*^2^ − 3 × 10^6^*x* + 4 × 10^6^, and the value of *R*^2^ is as high as 0.957.

We constrain the value of *A* to the range of 0 and 1. The formula of *A* is shown as follows:

14 $$A(n)=-\,4.048\times {10}^{-6}{t}^{6}+5.016\times {10}^{-3}{t}^{5}+0.015{t}^{4}+0.608{t}^{2}-3t+4$$

where *t* is defined as *n* mod *T* to represent an hour in a day, and *T* is the length of a period. In this paper, *t* is in hours and *T* is set to be 24.

## Footnotes

1 ^1^A serious explosion happened in China, see https://en.wikipedia.org/wiki/2015_Tianjin_explosions.

2 ^2^The most popular APP of news reading in China.
